# Perceived participation and autonomy structural relationships among related factors in patients with stroke and hypertension in China: A ISM model approach

**DOI:** 10.3389/fpubh.2022.1070998

**Published:** 2023-01-11

**Authors:** Le-ping Wan, Guang-mei Yang, Hai-ying Dong, Xiao-xiao Liang, Yan He

**Affiliations:** College of Public Health, Zhengzhou University, Zhengzhou, China

**Keywords:** perceived participation and autonomy, stroke, hypertension, ISM model, structural relationships

## Abstract

**Aims:**

To explore the structural relationship between perceived participation and autonomy among older adults with stroke and hypertension in home and community-based services (HCBSs) in the eastern coastal region of China.

**Design:**

An explorative cross-sectional study.

**Methods:**

From July to September 2021, a total of 714 respondents were reported to have stroke and hypertension, and their information was used in the analysis of this study. A multiple linear regression analysis was used to explore the factors influencing factors older adults' perceived participation and autonomy. Using the ISM model, we analyzed the factors affecting social participation in patients with stroke and hypertension and explained the logical relationships and hierarchy among the factors.

**Results:**

The mean score of perceived participation was 58.34 ± 27.57. Age, marital status, health insurance, living status, number of children, chronic diseases, sleep time, frequency of outings, and health utility value were significant factors affecting perceived participation and autonomy with stroke and hypertension patients. Among them, health insurance is the direct factor on the surface, age, number of children, chronic diseases, sleep time, frequency of outings, and health utility value are the intermediate indirect factors, and marital status and living status are the deep-rooted factors.

**Conclusion:**

By the study that the hierarchical structure provides a visualization of interrelationships and interdependences among the influencing factors of perceived participation and autonomy. It also may be a significant complement to traditional variable-entered approaches and construct an optimized multidimensional perspective of participation and autonomy. Future research should focus on optimizing the living environment of older adults with stroke and hypertension to explore the model of rehabilitative intervention and help patients successfully reintegrate into their families/societies.

## Introduction

Given the aging of the global population, the prevalence of various chronic diseases has increased significantly worldwide. Studies have shown that cardiovascular diseases have replaced malignant neoplasms as the leading cause of death in the population, and hypertension, as a major risk factor for cardiovascular diseases, with stroke as a typical example (1), and has attracted great attention worldwide (2, 3). China, an “aging giant,” has approximately 264 million people over 60 years old (18.7%) and 191 million people over 65 years old (13.5%) (4), and the prevalence of hypertension was as high as 53.2% of people over 60 years of age. Unlike hypertension, which takes years to manifest clinically, stroke presents sudden and devastating neurological consequences. Stroke is among the most common causes of death and disability worldwide (5), it can affect the afflicted person's functioning and cause the individual to experience chronic disability (6). Many people with stroke are not able to resume their previous roles in life or engage in various activities as they did in the past (7). As announced by the Chinese Center for Disease Control and Prevention, up to 2018, the number of individuals surviving a stroke in China had reached 12 million and approximately three-quarters of these survivors had dysfunctions, resulting in a social and economic burden of over 40 billion RMB per year (8). Their participation in their everyday lives can be markedly restricted after stroke and hypertension (9–13).

Arguably, one of the most important components of healthy aging is a person's participation in social life (14). In the International Classification of Functioning, Disability and Health (ICF), participation is defined as involvement in a life situation (15, 16). Autonomy is a prerequisite for effective participation. Autonomy means self-rule and implies that people have the right to make their own choices and decide how, when, and where to participate in activities (17). Thus, the concepts of participation and autonomy are strongly connected. The ability to participate autonomously to a sufficient extent can determine the health status of the elderly for some time. Cardol et al. (17) developed the Impact on Participation and Autonomy Questionnaire (IPA) to assess rehabilitation interventions and individual perceived impairment. The questionnaire has been translated into multiple languages (18), and the validity and reliability of IPA have been validated in Chinese populations and are continuously used to determine the quality of life in stroke patients (19, 20), spinal cord injury patients (21), and the elderly population (22). This study investigated the autonomous participation of the elderly using the revised Chinese version of the Impact on Participation and Autonomy Questionnaire by He et al. (23). An optimized multidimensional perspective of social participation is constructed by using the ISM model as a significant complement to traditional variable-entered approaches. The ISM model is a method of analysis developed by Professor Warfield, USA, which emphasizes the establishment of a hierarchical model of a complex system based on the relationships between system factors to determine the intrinsic correlation and hierarchy between the factors in the system (24, 25).

Based on large databases in China, national surveys (26, 27) and a survey of representative cities in some regions about social participation have been carried out, but little is known about social participation among older adults on the east coast of China. Based on the questionnaire data of home and community-based services (HCBSs) older adults in the east coastal area of China, this study uses a multiple linear regression analysis of the influencing factors of perceived participation and autonomy structural relationships among related factors in patients with stroke and hypertension and then further analyzes the correlation and hierarchical structure between the influencing factors through an ISM model to explore the internal mechanism affecting the participation and autonomy.

## Materials and methods

### Source of data

**Figure 1:**
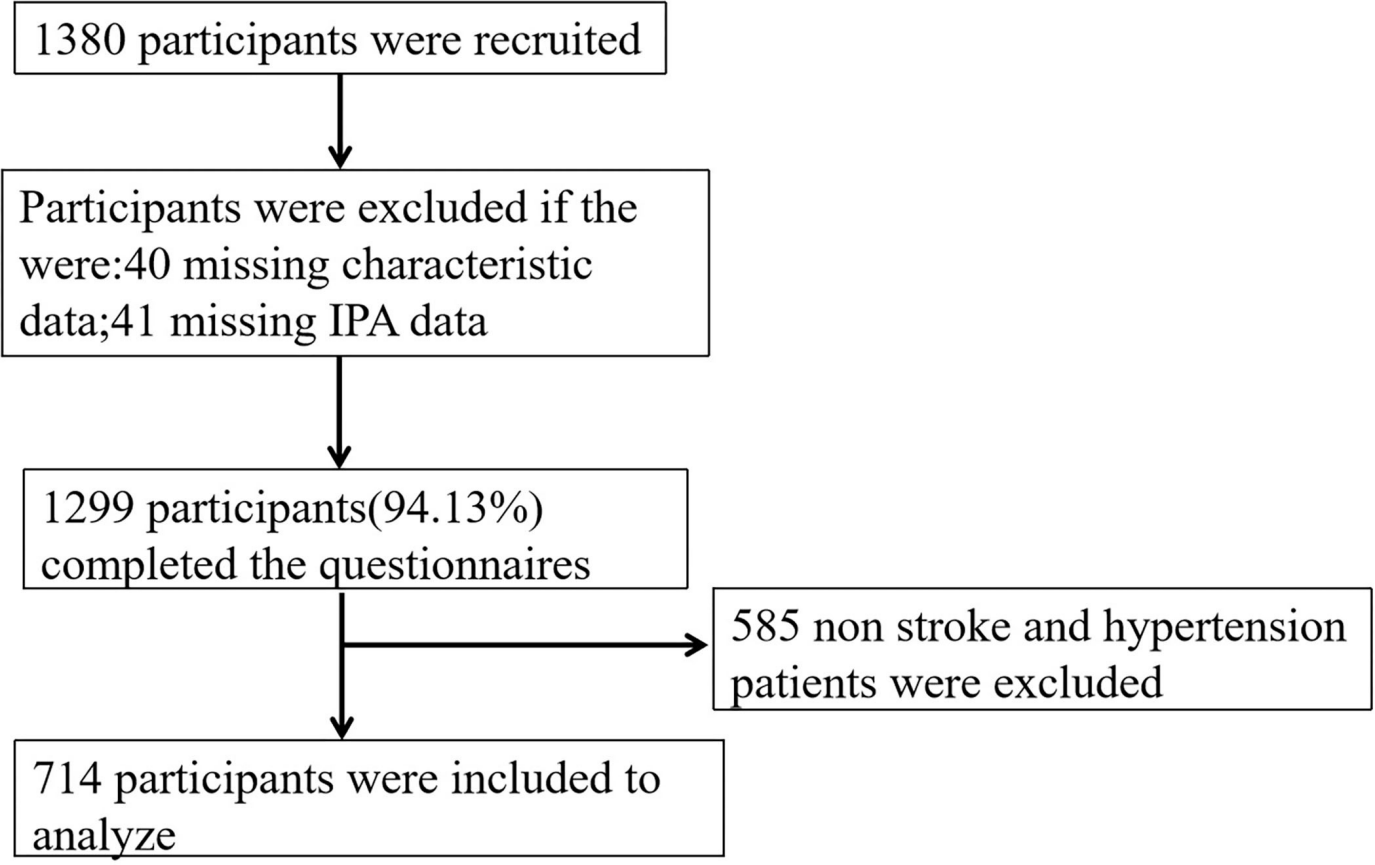
Flowchart on participant recruitment.

Based on the level of economic development and geographical location, China as a whole can be divided into three major economic regions, namely the eastern region, which is the first province to adopt the coastal opening policy and has a high level of economic development, the central region, which is the second most economically developed region, and the western region, which is the less economically developed region. In this study, the eastern region, which has a high level of economic development, is chosen as the target. Using cross-sectional investigation was conducted from September 2021 to December 2021 by the public health staff in the household survey. A multistage stratified whole-group sampling was used for this study design involving three provincial cities, namely, Suzhou, Guangzhou, and Qingdao, to obtain a representative sample of community-based adults aged 60 years or older. These provincial cities are by geographical location and socioeconomic development status. A county/district was randomly selected within the jurisdiction of each city. Two streets were randomly selected from each county/district. Therefore, Qingdao city was selected as Jimo district, Suzhou city was selected as Kunshan city, and Guangzhou city was selected as Yuexiu district. Finally, four communities were randomly selected from each street. Therefore, a total of 1,380 questionnaires were collected, missing values of key variables were excluded from the data, and 1,299 valid questionnaires were recovered. Elderly people with stroke and hypertension were screened out, and 714 participants were finally included (Figure 1).

### Impact on participation and autonomy questionnaire

Cardol and his colleagues (28) first developed the IPA to assess the restriction in participation and autonomy. The Chinese version of the IPA (IPA-C) revised by He et al. (23) was used in this study. The IPA-C comprises 25 items belonging to four domains: autonomy indoors (seven items), social relations (six items), family role (seven items), and autonomy outdoors (five items). The rating of the IPA-C is on a five-point Likert scale, with anchors at 0 (perfectly in line) to 4 (completely out of line). A lower score on the IPA-C indicates better self-perceived engagement and autonomy. The IPA-C has been previously used in various populations including stroke and hypertension patients, with reported Cronbach's α values of each domain between 0.81 and 0.985 (29, 30). Cronbach's α value in this study was 0.950.

### Statistical analyses

Descriptive summary statistics were estimated for sociodemographics, perceived participation, and autonomy. Means and standard deviations or medians with interquartile ranges were used for numerical variables, depending on their symmetry. The Interpretative Structural Modeling Method (ISM) is a method developed by Professor Warfield in 1976 to analyze complex analytical methods. Through the ISM model, we can obtain the multi-level hierarchical structure among the elements of the complex system, make many staggered elements organized and clear, and find the internal relationship between the main elements, so as to provide a reference for revealing the internal law of system structure and extracting useful information (31, 32). This study used *S*_0_ to indicate the social participation ability of community-dwelling older adults with stroke or hypertension, and *S*_*i*_ (*i* = 0, 1, ..., k) *S*_*j*_ to indicate the influencing factors affecting their willingness to implement the behavior. The elements in the adjacency matrix R can be determined by Equation (1):


(1) $$R=\{\begin{array}{c} 1,\;S_{i}\;is\;related\;to\;S_{j}\\ \;0,\;S_{i}\;is\;not\;related\;to\;S_{j} \end{array}\;$$

(2) $$\begin{array}{l} \text{i}=0,1,\ldots \ldots k;j=0,1,\ldots \ldots k \end{array}$$

The adjacency matrix of these factors can be obtained. The adjacency matrix reflects the direct relationship between elements, the reachability matrix reflects the indirect relationship between elements, and the reachability matrix between factors can be obtained from Formula (2):


(3) $$\begin{array}{l} M=(R+I)\lambda +1=(R+I)\lambda \neq (R+I)\lambda -1\neq \cdots \\ \;\neq (R+I)2\neq (R+I) \end{array}$$

In Formula (2), *R* is the original adjacency matrix, *I* is the identity matrix 2 ≤ λ ≤ k. The power operation in the matrix adopts the Boolean algorithm.

The factors contained in the top layer can be determined according to Formulas (3) and (4):


(4) $$\begin{array}{l} \text{P}\left(S_{i}\right)=\left\{S_{j}|m_{ij}=1\right\},\text{Q}\left(S_{i}\right)=\left\{S_{j}|m_{ji}=1\right\} \end{array}$$

(5) $$L_{1}=\left\{S_{j}|P(S_{i})\cap Q(S_{j})=P(S_{i});i=0,1,2\cdots ,k\right\}$$

In Formula (3), *m*_*ij*_ and *m*_*ji*_ refer to the factor of reachability matrix M. *P*(*S*_*i*_) is the reachable set, representing the set of all reachable factors starting from *S*_*i*_ in the reachable matrix; *Q*(*S*_*i*_) is the antecedent set and represents the set of all factors that can reach factor Si in the reachable matrix.

## Results

### Characteristics of the participants

A total of 714 patients were participating in the study (see Table 1) with a response rate of 85.5%. Their average age was 76.96 (SD 9.26). Most were men (55.0%), married (70.4%), and with co-morbid diseases (69.1%). Approximately half of the participants (82.6%) were cared for by their families and 2.8% of them with caregivers.

**Table 1:** Characteristics of participants and comparison of IPA scores among stroke survivors with different characteristics and expected direction.

**Variable**		***N* (%)**	**Total IPA**		
			**Mean (SD)**	**Statistics**	**Expected direction**
GenderX_1_	Male	321 (45.0)	58.21 (1.54)	102.32	**±**
	Female	393 (55.0)	58.45 (1.40)		
Age X_2_	*M(SD)*	*NA*	*NA*	377.49^**^	**+**
Marital status X_3_	Married	503 (70.4)	52.71 (1.18)	141.39^**^	**±**
	Divorce/Widow	211 (29.6)	71.51 (1.87)		
Education X_4_	Primary school or below	454 (64.4)	62.90 (1.38)	308.66^*^	**+**
	Middle school	135 (19.1)	49.85 (2.03)		
	Post-secondary or tertiary	98 (13.9)	49.89 (2.05)		
	Bachelor's degree and above	18 (2.6)	54.11 (5.32)		
Health insurance X_5_	Basic employee health insurance	209 (29.3)	54.11 (1.68)	220.00^*^	**+**
	Basic resident health insurance	412 (57.7)	59.68 (1.47)		
	Other	93 (13.0)	61.42 (2.72)		
Living status X_6_	Alone	177 (24.8)	65.17 (2.02)	526.07^**^	**±**
	With family	413 (82.6)	49.01 (1.23)		
	With caregivers	20 (2.8)	86.79 (3.98)		
	Others	104 (14.6)	81.25 (2.24)		
Number of children X_7_	0	50 (7.0)	69.69 (4.23)	333.45^*^	**±**
	1	170 (23.9)	54.96 (2.28)		
	2	282 (39.7)	55.96 (1.55)		
	≥3	809 (29.4)	61.70 (1.95)		
Number of Visits X_8_	Everyday	244 (34.6)	46.90 (1.52)	719.62^**^	–
	One month	402 (57.0)	65.29 (1.37)		
	Three months	39 (5.5)	57.84 (5.06)		
	One year and above	20 (2.8)	59.53 (6.92)		
Monthly income X_9_	≤ 1,000	248 (35.4)	60.56 (1.76)	338.64^*^	**+**
	1,001–3,000	190 (27.1)	53.20 (2.19)		
	3,001–5,000	184 (26.2)	62.81 (1.94)		
	≥5,001	79 (11.3)	52.35 (2.59)		
Chronic diseases X_10_	*M(SD)*	*NA*	*NA*	214.80^*^	**+**
Smoking X_11_	Yes	520 (72.8)	57.21 (1.91)	94.07	–
	No	194 (27.2)	58.62 (1.25)		
Alcohol X_12_	Yes	141 (19.7)	47.11 (1.86)	105.34	–
	No	573 (80.3)	60.93 (1.20)		
Sleep time X_13_	< 6	225 (31.5)	57.22 (1.76)	233.62^*^	–
	6–8	324 (45.4)	56.61 (1.47)		
	>8	165 (23.1)	62.85 (2.56)		
Frequency of outings X_14_	Multiple times daily	306 (42.9)	38.88 (0.94)	666.09^**^	**+**
	Once per week or more	166 (23.3)	59.03 (1.72)		
	Once a month and more	68 (9.5)	75.26 (2.32)		
	Longer time	173 (24.3)	86.74 (1.76)		
Health utility value X_15_	*M(SD)*	*NA*	*NA*	7,679.20^**^	**±**

In the expected impact, “+” indicates that it may be a positive impact, “–” indicates that it may be a negative impact, “**±**” indicates that the impact is unknown.

M, mean; N, number; SD, standard deviation.

^*^Correlation is significant at 0.05 level.

^**^Correlation is significant at 0.01 level.

### Analysis of factors influencing the IPA of survey respondents

The analysis of the factors influencing the IPA of the survey respondents is shown in Table 2, and the other univariate analysis results are presented in Table 1. The total IPA was used as the dependent variable, and the factors that were statistically significant in the univariate analysis were used as independent variables for multiple linear regression analysis using the stepwise method. The accuracy of the model prediction was 73.7%, indicating that the prediction effectiveness was good. Overall, this regression model has a sound fit, strong explanatory power, predictive power, and high confidence in the regression results. In the final predicted results, Age (S1), Marital status (S2), Health insurance (S3), Living status (S4), Number of children (S5), Chronic diseases (S6), Sleep time (S7), Frequency of outings (S8), and Health utility value (S9), these nine factors had significant effects on the social participation of the respondents.

**Table 2:** Multiple linear regression analysis of perceived participation and autonomy of community homebound older adults.

**Variable**	**Model 1**				**Model 2**				**Model 3**			
	**B**	**SE**	**EXP(B)**	**T**	**B**	**SE**	**EXP(B)**	**T**	**B**	**SE**	**EXP(B)**	**T**
GenderX_1_	−0.03	0.98	−0.02	−0.86	−0.02	0.64	−0.01	−0.48	−0.02	0.96	−0.01	−0.63
Age X_2_	3.96	0.72	0.12	5.52**	4.67	0.75	0.14	6.21**	4.67	0.75	0.14	6.21**
Marital status X_3_	4.20	1.29	0.07	3.27**	4.76	1.29	0.08	3.68**	4.74	1.29	0.08	3.68**
Education X_4_	0.18	−0.05	−0.03	−1.33	−0.06	0.12	−0.03	−1.54	−0.07	0.09	−0.04	−1.69
Health insurance X_5_	0.05	0.07	0.04	2.01*	0.07	0.08	0.04	1.76	0.03	1.72	0.07	0.97
Living status X_6_	0.04	0.90	0.05	2.13*	0.06	0.77	0.04	1.56	0.07	0.76	0.04	1.877
Number of children X_7_	−0.09	0.84	−0.6	−2.82*	−1.92	0.68	−0.61	2.82**	−1.85	0.68	−0.06	2.73**
Number of Visits X_8_	0.05	0.94	0.03	1.28	0.02	0.53	0.01	0.63	0.16	0.88	0.09	0.42
Monthly income X_9_	0.03	0.98	0.02	1.12	0.04	0.04	0.02	0.97	0.03	0.43	0.16	0.79
chronic diseases X_10_	0.07	0.96	0.04	1.93	0.07	0.95	0.04	1.83	0.06	0.14	0.03	1.46
Smoking X_11_	0.03	0.99	0.02	1.01	0.03	0.52	0.01	0.65	0.21	0.97	0.11	0.55
Alcohol X_12_	−0.07	0.96	−0.03	−1.35	−0.06	0.94	−0.03	−1.55	−0.06	0.12	−0.03	−1.22
Sleep time X_13_	−0.09	0.98	−0.05	−2.33*	−0.08	0.98	−0.04	−2.21	−1.63	0.74	−0.04	−2.21*
Frequency of outings X_14_	7.15	0.58	0.32	12.30**	6.84	0.59	0.30	11.54**	6.84	0.59	0.30	11.54**
HRQoL X_15_	−52.74	2.44	−0.55	−21.65**	−53.38	2.43	−0.56	−22.11**	−53.69	2.43	−0.56	−22.11**
R	0.858				0.860				0.861			
F	473.342				384.158				322.793			
Prob > chi^2^	< 0.001				< 0.001				< 0.001			
Model predictive accuracy	73.5%				73.7%				83.9%			

β, standardized coefficients.

^*^Correlation is significant at 0.05 level.

^**^Correlation is significant at 0.01 level.

### ISM regression

**Figure 2:**
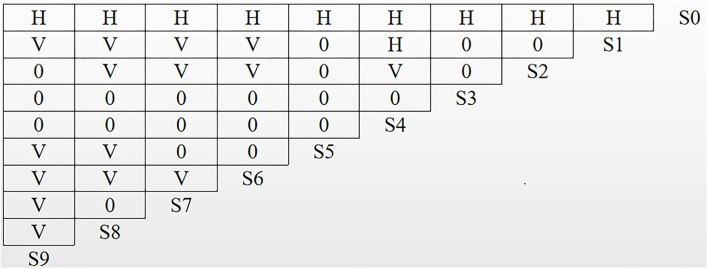
Logical relationships of influencing factors.

To further examine the logical association and hierarchical structure among the factors, ISM was performed on the influencing factors using MATLAB 8.0 software. In Figure 2, H indicates that the column factors have a direct or indirect influence on the row factors, V indicates that the row factors have a direct or indirect influence on the column factors, and 0 indicates that there is no mutual influence between the factors.

**Figure 3:**
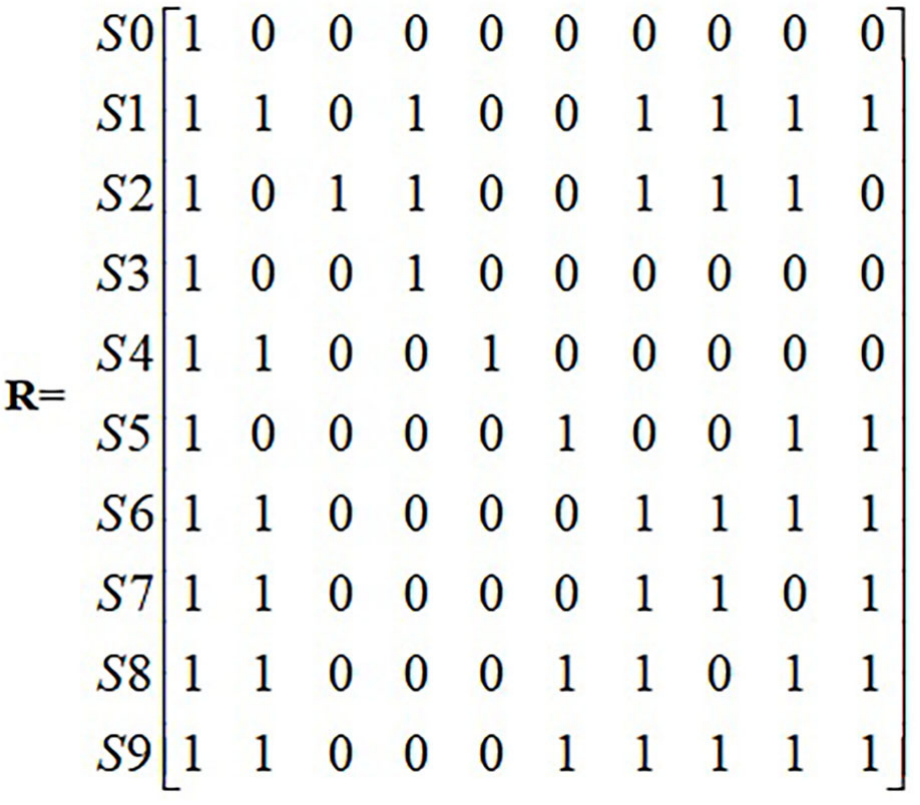
Adjacency matrix.

Based on the logical relationship between the influencing factors, the corresponding adjacency matrix R is generated according to Equation (1) (Figure 3).

**Figure 4:**
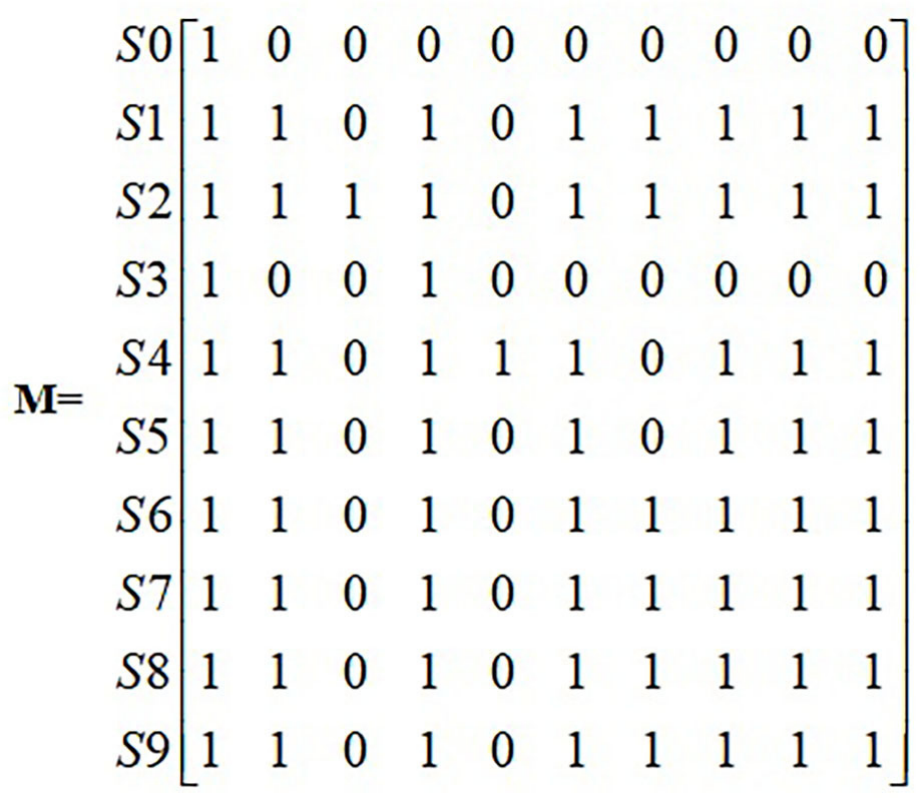
Reachability matrix.

The adjacency matrix R is substituted into the software Matlab 7.0 to calculate the reachable matrix M (Figure 4).

**Figure 5:**
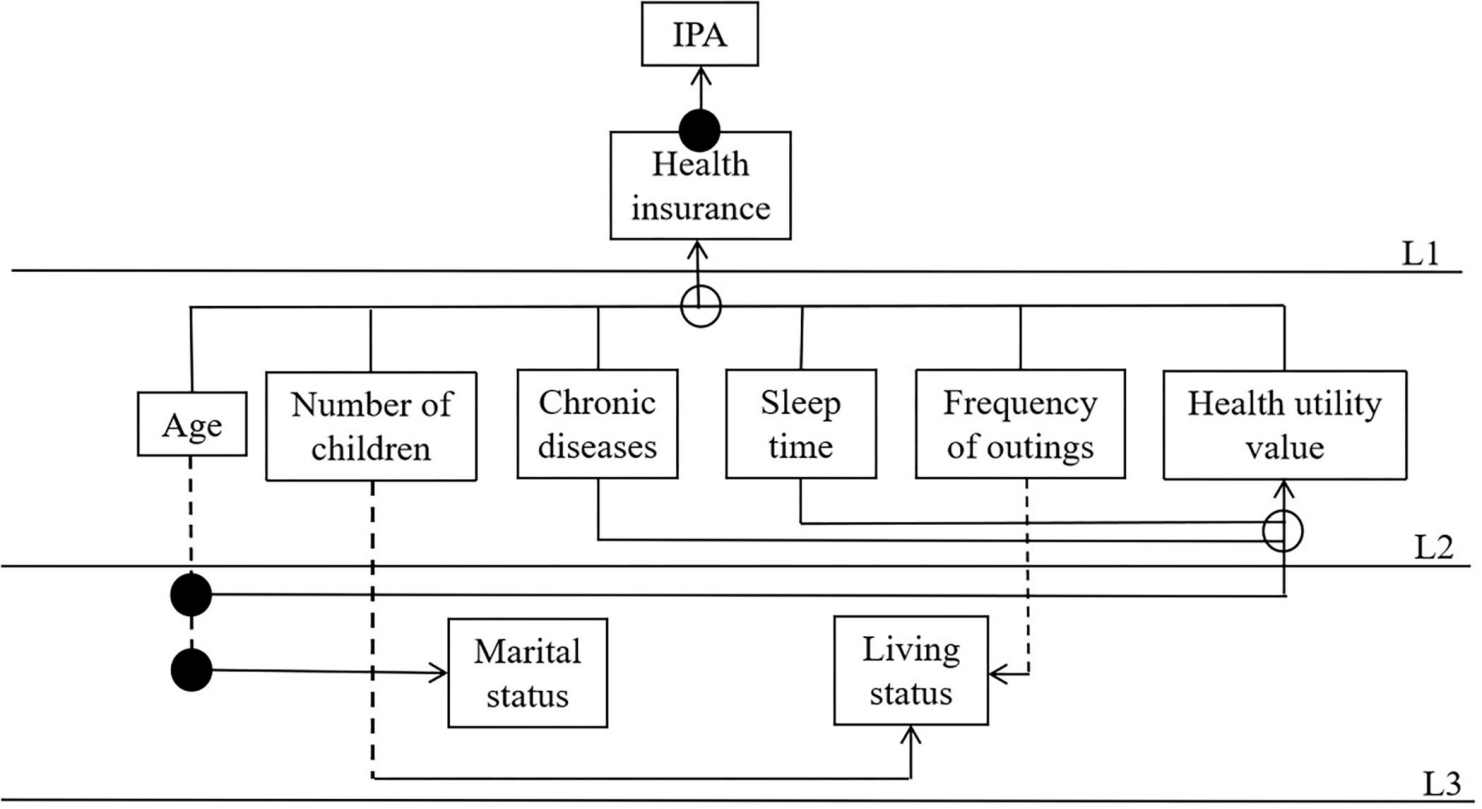
Interpretative structural model of influencing factors. A solid line indicates direct influence, a dashed line indicates cross-level influence, a solid dot indicates two related influence lines, and a hollow dot indicates three or more influence relationships.

The reachability matrix was decomposed according to formulas (3) and (4) to obtain the factor layers composed of different influencing factors and to determine the hierarchical relationships. We obtained Figure 5 in order to make the relationship between factors more intuitive.

According to the results of MATLAB calculations, the nine significant factors influencing perceived participation and autonomy are divided into three levels: the first layer includes health insurance (S3); the second layer includes age (S1), number of children (S5), chronic diseases (S6), sleep time (S7), frequency of outings (S8), and health utility value (S9); and the third layer includes marital status (S2) and living status (S4). Through sorting, the correlation and hierarchy among the influencing factors of patients with stroke and hypertension among the influencing factors of perceived participation and autonomy, in China, are obtained as shown in Figure 5.

## Discussion

Based on the data obtained by the research group and by establishing the ISM model, this article studies the influencing factors of perceived participation and autonomy by 714 older adults patients with stroke and hypertension. The hierarchical structure provides a visualization of interrelationships and interdependences among the influencing factors of perceived participation and autonomy. It can serve as a useful reference for perceived participation and autonomy.

By integrating the results of the studies in Table 2 and Figure 5, it is shown that among the influential factors affecting patients' perceived participation and autonomy, among them, health insurance is the direct factor on the surface, age, number of children, chronic diseases, sleep time, frequency of outings, and health utility value are the intermediate indirect factors, and marital status and living status are the deep-rooted factors. Management priority and preventive measures should be arranged according to the hierarchy level. The rule is that the higher the hierarchy level, the more attention is deserved. Direct factors on the surface in L1 are the most fundamental and important factors. Transition factors in L2 connect the root cause layer and the direct cause layer in the system. Marital status and living status are the direct causes.

According to an assessment of the direct factors on the surface, the type of health insurance had a significant positive effect on patients' perceived participation and autonomy, in accordance with the expected hypothesis. In the course of disease treatment, the patient's type of health insurance determines the percentage of reimbursement for the cost of the visit and was closely related to the vital interests of the elderly. The results of the research by He et al. (33) also indicated that there was an effect of economic status on the perceived participation and autonomy of older adults, which was consistent with the results of this study. The low rate of health insurance means that poor patients are not compensated for treatment and rehabilitation costs. This suggests that society should pay more attention to the social participation of the low-income elderly population and increase reimbursement rates to ensure that patients can undergo rehabilitation (34).

According to the examination of intermediate indirect factors, age, number of children, chronic diseases, sleep time, frequency of outings, and health utility value were the middle-level indirect influencing factors. Among them, age, chronic diseases, and frequency of outings factors had a positive effect on the dependent variable, and number of children, sleep time, and health utility value had a negative effect. Chau et al. (35) found age to be among the factors that had a direct effect on participation restrictions among the Asian sample of persons with stroke. A decline in participation was found with an elder age, which is in accordance with Ang (36) and Gao et al. (37) studies. As older adults age, they gradually lose control over their mental and social resources, become increasingly limited in their daily activities, experience an increasing amount of events beyond their capabilities, and have a diminishing opinion of their own abilities (38). This result is consistent with Wang et al. (39). Older adults with chronic diseases generally have a residual degree of physical, psychological, and cognitive impairment and changes in family status roles, all of which can lead to social avoidance, a reluctance to communicate with them, less frequent outings, and reduced social activities (40). Furthermore, Fallahpour et al. (41) results emphasize the importance of physical function, mood state, and access to caregiving services as predictors of participation in everyday life after stroke and hypertension. This suggests that families, friends, and relatives should often care for patients, give them the opportunity to complain, provide emotional help and support, and improve their subjective social support, which in return improves their autonomous participation in social life (42).

According to an assessment of deep-rooted factors, it can be found that the root cause factors set of marital status and living status, which are at the bottom of the system and are not affected by other factors, can directly or indirectly affect other factors and result within the system, which should be paid more attention. Older adults whose spouses were alive or who lived with their spouses or children had higher self-efficacy, lower loneliness, and higher social participation. This result suggests that widowhood is a serious negative life event for older adults and can significantly affect their physical and psychological health. Cultural differences regarding family orientation might be one explanation since China can be regarded as being a very family-oriented society with clear roles within the family and distinct expectations with respect to care and support within the family context (43), all of which shape and reflect the social environment.

## Limitations

The research has some limitations that need to be addressed in future work. First, the current study is a cross-sectional descriptive study, limiting its ability to make a causal argument. A longitudinal study is needed to further investigate the interaction between health factors and social participation. Second, in terms of variable selection and validation, the fact that some indicators failed the significance test does not mean that they have no effect on perceived participation and autonomy, and follow-up studies may consider choosing other models to analyze the influencing factors of perceived participation and autonomy from multiple perspectives. There are 11 provinces in eastern China, and we only selected three provinces and cities with representative characteristics, including Suzhou, Guangzhou, and Qingdao. More provinces and people in eastern China will be included to increase the representativeness of the sample in future research. Finally, we cannot rule out confounders because of certain unmeasured parameters that may influence social participation and level changes.

## Conclusion

This study revealed the three-hierarchy structure of factors influencing perceived participation and autonomy in patients with stroke and hypertension, with health insurance is the direct factors on the surface, age, number of children, chronic diseases, sleep time, frequency of outings, and health utility value are the intermediate indirect factors, and marital status and living status are the deep-rooted factors. It is important that family physicians promptly identify low levels of social participation and implement intervention measures aimed at confronting the factors negatively affecting perceived participation and autonomy. Further studies are required to explore the model of rehabilitative intervention.

## Data availability statement

The original contributions presented in the study are included in the article/supplementary material, further inquiries can be directed to the corresponding author.

## Ethics statement

The study was approved by Zhengzhou University Life Science Ethics Committee (Code: ZZUIRB2022-07) and was conducted following the principles of the Declaration of Helsinki.

## Author contributions

L-pW designed this study, participated in its implementation and coordination, and helped draft the manuscript. G-mY performed the statistical analysis. H-yD and X-xL helped collect the data. YH helped with the formatting of this manuscript. All authors have seen and approved the final manuscript.
